# Effects of pH and Temperature on the Structure and Function of Pea Albumin

**DOI:** 10.3390/molecules31020381

**Published:** 2026-01-21

**Authors:** Xinxin Li, Guozhi Ji, Bingyu Chen, Wenhui Li, Xiaomin Li, Jie Liu, Zhishen Mu, Ziyuan Wang, Hongzhi Liu

**Affiliations:** 1 Key Laboratory of Geriatric Nutrition and Health, Beijing Technology and Business University, Ministry of Education, Beijing 100048, China; 2 Inner Mongolia Enterprise Key Laboratory of Dairy Nutrition, Health & Safety, Hohhot 011500, China; 3 Global R&D Innovation Center, Inner Mongolia Mengniu Dairy (Group) Co., Ltd., Hohhot 011500, China

**Keywords:** pea protein albumin, structure, function, pH-shifting, temperature, zeta potential, protein stability, functional properties

## Abstract

Pea albumin is a high-quality plant-based protein with growing relevance in food applications, yet the effects of pH and thermal treatment on its structural and functional properties remain insufficiently understood. This study investigated the effects of environmental factors, namely pH (3, 5, 7, 9) and temperature (40, 60, 80, 100 °C), on the structural behavior and functionality of pea albumin. Structural changes were characterized through particle size, Zeta potential, surface hydrophobicity, and intrinsic fluorescence. Functional properties, including solubility, foaming ability, and emulsifying capacity, were evaluated and compared with untreated controls. Under alkaline conditions (pH 9), stronger electrostatic repulsion led to a 29.5% reduction in particle size, a 76.47% increase in Zeta potential, enhanced protein unfolding, and a 19.06% increase in surface hydrophobicity. At this pH, solubility increased by 24.8%, accompanied by notable improvements in foaming and emulsifying performance. Moderate heating (40, 60 °C) induced partial unfolding, resulting in decreased particle size and enhanced solubility, which further contributed to improved functional behavior. Pearson correlation analysis demonstrated significant associations between structural indicators (particle size, Zeta potential, surface hydrophobicity) and functional properties, highlighting the structure–function relationship of pea albumin. This work provides a comprehensive understanding of environmental factor-induced changes in pea albumin and offers valuable insights for its optimized application in plant-based foods.

## 1. Introduction

With increasing awareness of climate change and the continuous growth of the global population, the sustainable development of food systems has become a central concern worldwide. Traditional animal-based diets are considered ecologically burdensome; therefore, the development of alternative protein sources derived from renewable plant resources has become an inevitable trend [1,2]. Moreover, as soy and wheat are among the most common food allergens [3], legume proteins such as those obtained from peas, mung beans, and faba beans have attracted growing attention owing to their low allergenicity and well-balanced nutritional composition. Their potential application in functional and plant-based foods has been increasingly recognized in recent years.

Pea is an important leguminous crop that has long been used as both human food and animal feed. In terms of global edible legume production, pea ranks fourth, following soybean, peanut, and common bean. Pea seeds contain approximately 23.1–30.9% protein, 50–60% carbohydrates, 1.5–2% lipids, and small amounts of minerals, vitamins, polyphenols, and phytic acid [4]. Owing to its favorable gelling, emulsifying, and foaming properties, pea protein has been recognized as a valuable ingredient for food formulation [5]. Based on solubility, pea protein can be divided into water-soluble albumins and salt-soluble globulins, with albumins accounting for 15–25% and globulins for 55–80% of the total protein fraction [6,7]. Pea albumin consists mainly of two components, PA1 and PA2, which contribute approximately 50% and 16% of the sulfur-containing amino acids in pea seeds, respectively. Recently, plant-derived albumins have been reported to exhibit better technological functionality than globulins, particularly in terms of solubility, foaming, and emulsifying behavior [8]. Although globulins are the most abundant proteins in peas, we focused on albumins due to their distinct functional properties. Albumins are highly water-soluble, have lower molecular weight, and exhibit superior foaming and emulsifying performance compared with globulins. Furthermore, albumins have been less extensively studied, and thus investigating their structure–function relationship helps to fill a knowledge gap in pea albumin research.

Environmental factors such as pH, temperature, and prolonged storage are known to affect the structural conformation and functional behavior of proteins. Among these factors, pH and temperature are considered particularly influential [9]. It has been reported by Tanger et al. that the gelation of pea protein is affected by pH through changes in denaturation and solubility [10]. It was shown by Othmeni et al. that pH-induced variations in electrostatic interactions can alter the Zeta potential, particle size, and surface tension of protein dispersions, which affects their stability [11]. In addition, it was found by Higa et al. that alkaline pH combined with heating can enhance protein solubility, while acidic pH with heating can reduce protein stability in solution [12]. Recent studies have also provided new insights into pea albumin itself. Li et al. [8] conducted an in-depth structural characterization of pea albumin and its fractions, demonstrating that thermal treatment induces distinct unfolding and aggregation behaviors that strongly influence foaming properties. Similarly, Li et al. investigated the structural evolution of pea-derived albumins under different pH and temperature conditions, showing that environmental factors can modulate their hierarchical structure from the nanoscale to the supramolecular level [13]. Collectively, these findings indicate that environmental conditions have significant effects on the structure and functionality of proteins. However, systematic studies on the structural changes and processing behavior of pea albumin under varying pH and temperature conditions remain scarce. In particular, the relationship between environmental factor-induced conformational transitions and corresponding functional properties has not been fully elucidated. Therefore, this study systematically investigates the combined effects of multiple pH and temperature conditions on pea albumin. By analyzing its structural stability and processing behavior, we provide a deeper understanding of the structure–function relationship of pea albumin. This work fills a critical knowledge gap in pea albumin research and has practical implications for the development of plant-based foods, where optimized solubility, foaming, and emulsifying properties are essential for improving texture and stability in products such as beverages, foams, and baked goods.

## 2. Results and Discussion

### 2.1. Sodium Dodecyl Sulfate–Polyacrylamide Gel Electrophoresis (SDS-PAGE)

**Figure 1:**
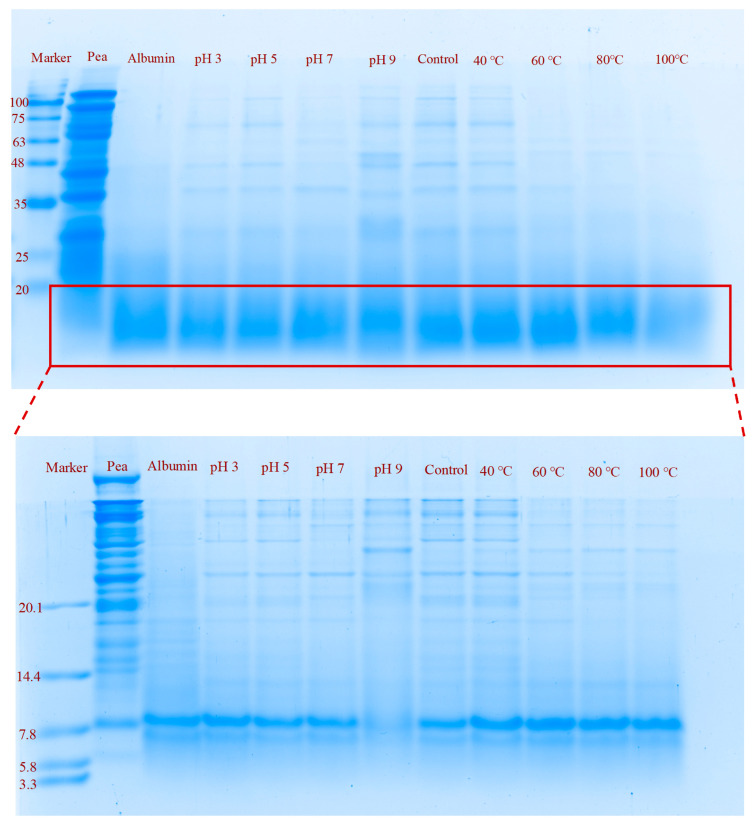
SDS-PAGE profiles of total pea protein and pea albumin subjected to different treatments under reducing conditions. Pea: total pea protein. pH 3, 5, 7, and 9: pea albumin treated at different pH conditions. 40, 60, 80, and 100 °C: pea albumin subjected to different heating temperatures.

Figure 1 shows the pea protein and reducing SDS-PAGE profiles of albumin subjected to different treatments. Overall, pea albumin was effectively isolated, with most protein bands distributed below 20 kDa. The dominant component was PA1. PA1 consists of two subunits, PA1a and PA1b [8], which can be clearly distinguished as two separate bands on the gel. Neither pH treatment nor thermal treatment caused marked changes in the subunit composition of pea albumin. However, under pH 5 conditions, some high-molecular-weight bands appeared, which is likely attributed to protein aggregation near the isoelectric point. In addition, high-temperature treatment induced partial aggregation of albumin, resulting in a decrease in the intensity of low-molecular-weight bands.

### 2.2. Particle Charge and Size

**Figure 2:**
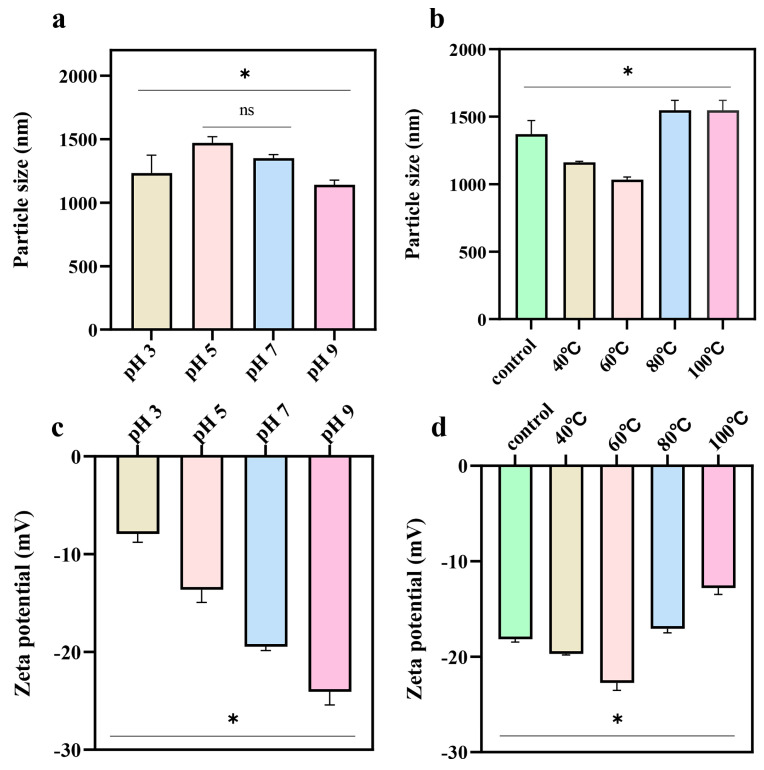
The particle size and Zeta potential of pea albumin under different pH conditions (3, 5, 7, and 9) and temperatures (40, 60, 80, and 100 °C), (*n* ≥ 3). (**a**) particle size variation with pH, (**b**) particle size variation with temperature, (**c**) zeta potential variation with pH, and (**d**) zeta potential variation with temperature. * indicates a significant difference between samples, *p* < 0.05; ns indicates no significant difference.

The effects of pH and temperature on the particle size and Zeta potential of pea albumin are presented in Figure 2. As shown in Figure 2a, the particle size of pea albumin initially increased and then sharply decreased as pH increased, reaching a maximum at pH 5, which was approximately 12% higher than that at pH 7 (control). This behavior can be attributed to enhanced protein aggregation near the isoelectric point (pI) of pea albumin [14]. At pH values close to the pI, the electrostatic repulsion between protein molecules is minimized, facilitating aggregation and resulting in larger particle sizes. In contrast, when the pH deviates from the pI, the net surface charge increases, leading to stronger electrostatic repulsion and consequently smaller particle sizes [15]. The tendency of proteins to aggregate is closely related to electrostatic repulsion between protein particles. This repulsion creates an energy barrier that limits their proximity and thereby inhibits aggregation [16]. The Zeta potential results further support this trend (Figure 2c). Compared with pH 5 (−13.6 mV), the absolute values of Zeta potential at pH 7 (−19.5 mV, control) and pH 9 (−24 mV) were substantially higher [17]. The greater the absolute value of the zeta potential, the more amino acid residues with the same charge appear on the protein surface. The results indicated that repulsive electrostatic interactions between groups enhanced under alkaline conditions.

As shown in Figure 2b, compared to control, moderate heating (40 and 60 °C) decreased the particle size of albumin. This treatment likely caused partial unfolding of the protein structure, which improved dispersion [18]. In contrast, excessive heating (80 and 100 °C) increased particle size. This suggests that high-temperature heating promotes stronger protein polymerization. High temperatures typically strengthen protein–protein interactions, leading to aggregation [16]. A similar trend was observed for Zeta potential (Figure 2d). Moderate heating (40 and 60 °C) increased the absolute value of the potential, likely due to the exposure of additional charged groups. However, excessive heating (80 and 100 °C) weakened the electrostatic repulsion between protein surfaces, resulting in dissolution and dispersion that could not overcome the aggregation and folding of molecules. These particles tended to aggregate within the system [19].

### 2.3. Secondary Structure

**Figure 3:**
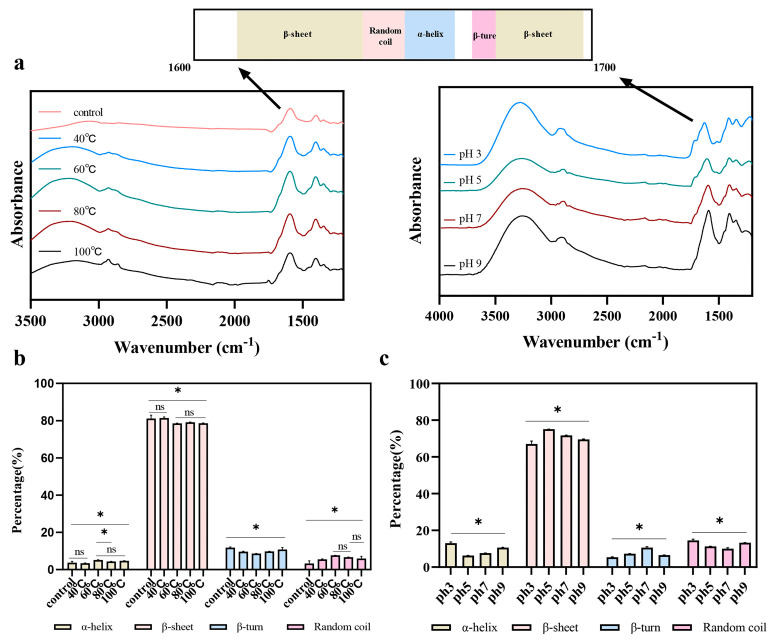
FTIR spectra and secondary structure compositions of pea albumin at different pH values (3, 5, 7 and 9) and temperatures (40, 60, 80, and 100 °C), (*n* ≥ 3). (**a**) fourier transform infrared (FTIR) spectra at different pH levels and temperatures, (**b**) secondary structure content at different pH levels, (**c**) secondary structure content at different temperatures. * indicate a significant difference between samples, *p* < 0.05; ns indicates no significant difference [24].

FTIR spectra of pea albumin at different pH levels (3, 5, 7, 9) and temperatures (40, 60, 80, 100 °C) are presented in Figure 3a. All samples exhibited characteristic absorption bands at 1700–1600 cm^−1^ (Amide I and C=O stretching vibration of the peptide linkage) [20]. Consistent with the findings of Othmeni et al. [11], pea albumin was predominantly composed of β-sheets and β-turns. As shown in Figure 3b, the secondary structure of pea albumin displayed pronounced pH-dependent variations. The ratios of the secondary structure of the proteins were obtained by fitting Gaussian linear function to the FTIR spectra in the region of 1700–1600 cm^−1^ [20]. The β-sheet content was highest at pH 5, and under this condition the solubility of pea albumin was also the lowest. Pearson correlation analysis further confirmed this relationship (Figure 7a), showing a significant negative correlation between β-sheet content and solubility (R^2^ = −0.77, *p* < 0.05). Similar trends have been reported previously, where proteins with a higher proportion of β-sheets were shown to possess more rigid molecular conformations and consequently exhibit lower solubility [21]. When the pH shifted from 5 to either 3 or 9, a notable decrease in β-sheet content occurred, the protein gradually unfolded [22]. In addition, the content of random coils increased under both acidic (pH 3) and alkaline (pH 9) conditions. This increase suggests that extreme pH environments promote partial unfolding of the albumin structure and disrupt its ordered secondary structure, resulting in a more flexible and loosely packed molecular conformation [17].

As shown in Figure 3c, compared to control, moderate heating caused a decrease in β-sheet content and an increase in α-helix content. This change may result from gradual structural stretching during heating, which promotes protein unfolding and disrupts hydrogen bonds between adjacent peptide chains. The breakdown of β-sheets, together with enhanced hydrophobic interactions, facilitates the formation of α-helices. Under excessive heating, the α-helix content decreased, while the β-sheets and β-turns contents increased. This pattern indicates heat-induced denaturation. High temperatures can break hydrogen bonds between carbonyl and amino groups, causing α-helical structures to unravel and transform into β-sheets or β-turns structures. These changes ultimately promote aggregation [23].

### 2.4. Surface Hydrophobicity (H_0_)

**Figure 4:**
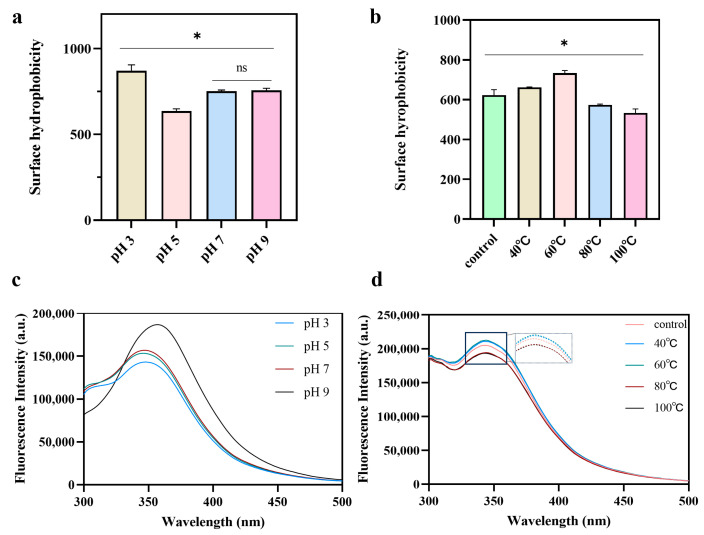
Changes in surface hydrophobicity and intrinsic fluorescence of pea albumin at different pH values (3, 5, 7, and 9) and temperatures (40, 60, 80, and 100 °C), (*n* ≥ 3). (**a**) surface hydrophobicity variation with pH, (**b**) surface hydrophobicity variation with temperature, (**c**) intrinsic fluorescence variation with pH, (**d**) intrinsic fluorescence variation with temperature. * indicate a significant difference between samples, *p* < 0.05; ns indicates no significant difference.

The Surface hydrophobicity (H_0_) is an indicator of the degree of exposure of hydrophobic groups in a protein molecule, which is closely related to the structural and functional properties of proteins. the effect of pH on the H_0_ of pea albumin is shown in Figure 4a. The lowest H_0_ value is found at pH 5, which may be due to being near the isoelectric point. The proteins near the isoelectric point are relatively aggregated and the hydrophobic groups are buried inside the protein, which leads to a decrease in H_0_ on the surface of the protein [25]. A significant increase in H_0_ was observed when the pH was decreased from 5 to 3, increased by about 37%. This may be related to the increase in the content of irregular curls in the protein structure, leading to the exposure of more hydrophobic groups, and a significant increase in H_0_ of proteins by acidic environments has also been observed in a previous report [22]. When pH was increased from 7 to 9, there was little change in H_0_, suggesting that a similar degree of hydrophobic groups were present within the protein. Alkaline environments may promote the deprotonation of amino acid side chains, increase electrostatic repulsion and facilitating partial unfolding of the protein, which in turn leads to the exposure of previously buried hydrophobic groups [15]. The effect of temperature on the surface hydrophobicity (H_0_) of albumin is presented in Figure 4b. Compared with the control, moderate heating resulted in an increase in H_0_ [26], indicating enhanced exposure of hydrophobic regions. Since ANS fluorescence intensity increases upon binding to exposed hydrophobic sites, the observed fluorescence enhancement at moderate temperatures provides evidence of partial protein unfolding. This unfolding exposes hydrophobic side-chain residues that were originally buried within the globular core of pea albumin, indicating changes in tertiary structure [27]. Compared to the control and moderate heating (40, 60 °C), excessive heating reduced H_0_. This reduction may be attributed to heat-induced aggregation, which can re-bury hydrophobic groups within protein aggregates [23].

### 2.5. Intrinsic Tryptophan Fluorescence Spectroscopy

The intrinsic fluorescence of proteins mainly arises from three aromatic amino acids: tryptophan, tyrosine, and phenylalanine. Among these, tryptophan plays the dominant role in determining the overall fluorescence intensity. The emission spectrum of the tryptophan indole chromophore is highly sensitive to the polarity of its surrounding environment [28]. All samples showed emission maxima near 330 nm, indicating that tryptophan residues were the main contributors to fluorescence [15]. As shown in Figure 4c, the fluorescence intensity of pea albumin increased when the pH was raised from 5 to 9. A clear red shift in λ_max_ was also observed. This suggests that the tryptophan residues are exposed to a more polar microenvironment. This exposure is likely caused by the unfolding of the pea albumin structure induced by pH treatment, which reveals previously buried chromophores and leads to an increase in fluorescence intensity [29]. As shown in Figure 4d, compared to the control, the fluorescence intensity of albumin shows no substantial changes with increasing temperature. Previous studies have reported that tryptophan residues can refold markedly when heated protein solutions are cooled to room temperature (25 °C) [30]. However, the partial spectral shifts observed here indicate that the local microenvironment does not fully return to its original state, or that not all protein molecules undergo complete refolding. Therefore, a slight increase in fluorescence intensity is observed under moderate heating, whereas excessive heating results in a slight decrease.

### 2.6. Solubility

**Figure 5:**
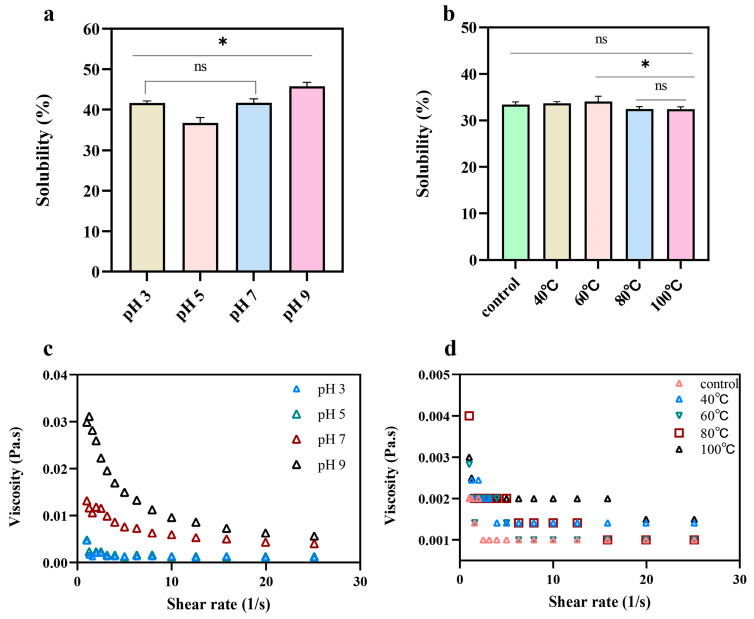
Changes in solubility and apparent viscosity of pea albumin at different pH values (3, 5, 7, and 9) and temperatures (40, 60, 80, and 100 °C), (*n* ≥ 3). (**a**) solubility variation with pH, (**b**) solubility variation with temperature, (**c**) apparent viscosity variation with pH, (**d**) apparent viscosity variation with temperature. * indicate a significant difference between samples, *p* < 0.05; ns indicates no significant difference [34,35].

**Figure 7:**
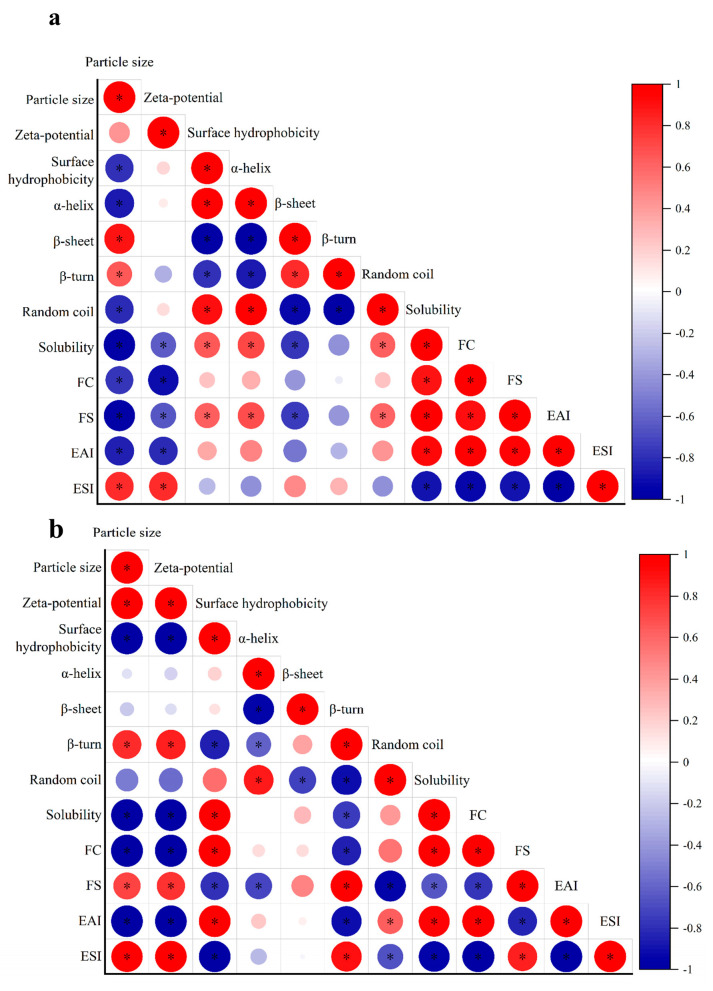
Pearson correlation analysis of structural characterization and functional properties of albumin at different pH values (3, 5, 7, and 9, (**a**)) and temperatures (40, 60, 80, and 100 °C, (**b**)). The color scale ranges from dark blue (strong negative correlation, −1) to white (no correlation, 0) and to dark red (strong positive correlation, 1). The parameters include particle size, zeta potential, surface hydrophobicity, α-helix, β-sheet, β-turn, random coil, solubility, FC, FS, EAI, and ESI. * indicates a significant correlation, *p* < 0.05.

Protein solubility is a key functional indicator, and its magnitude strongly affects other functional properties. As the pH increased from 3 to 9, the solubility of pea albumin exhibited a typical U-shaped trend (Figure 5a), consistent with previous reports [8]. The lowest solubility occurred at pH 5, where proteins tend to aggregate near their isoelectric point. When the pH moved away from this region, the solubility gradually increased. Compared with pH 5, the solubility increased by 13.35% at pH 3 and by 24.8% at pH 9. Pearson correlation analysis (Figure 7a) further indicated that solubility was significantly negatively correlated with particle size and Zeta potential (R^2^ = −0.97 and −0.65, respectively; *p* < 0.05). This improvement can be attributed to stronger electrostatic repulsion and smaller particle size, as proteins with higher net charges generally exhibit higher solubility [31].

The effect of temperature on the solubility of albumin is shown in Figure 5b. Compared to the control, solubility increased gradually with temperature, peaking at 60 °C before decreasing at 80 °C and 100 °C. This result is consistent with the findings of Yu et al. on the effect of heat treatment on the solubility of soy protein [32]. This change may be related to the particle size, with smaller particle size indicating an increase in the contact area between water and protein, which suggests that as the particle size decreases, the solubility increases with it, which is consistent with the previous trend of particle size change; whereas too high a temperature leads to enhanced denaturation of proteins, which triggers aggregation of proteins, resulting in an increase in the size of the particles, and hence a decrease in the solubility [33]. Pearson correlation analysis (Figure 7b) further confirmed a significant negative correlation between solubility and particle size (R^2^ = −0.99, *p* < 0.05).

### 2.7. Steady Shear

As shown in Figure 5c, all samples exhibited shear-thinning behavior, characterized by a decrease in apparent viscosity with increasing shear rate, consistent with previous reports [36]. This behavior may be attributed to the increase in shear rate, which causes deformation or rearrangement of protein particles and consequently reduces flow resistance [37]. The higher apparent viscosity observed at pH 9 may be due to the stronger negative charge on albumin molecules. This is consistent with the results for the absolute value of the Zeta potential under alkaline conditions. The enhanced electrostatic repulsion increases intermolecular spacing and contributes to greater resistance to flow [14]. The influence of temperature on apparent viscosity agrees with the results reported by Zhang et al., demonstrating that heating treatments consistently enhance the apparent viscosity of albumin [38]. The viscosity reached its maximum at 100 °C. This can be attributed to enhanced protein–protein interactions and the formation of larger aggregates. When an external force is applied, these aggregates generate greater resistance to flow, resulting in higher apparent viscosity [39]. This increase may be related to intermolecular hydrophobicity-driven cross-linking, and may also be partially attributable to certain hydrogen bond changes and physical entanglement induced between proteins during the heating process [40,41].

### 2.8. Foaming Properties

**Figure 6:**
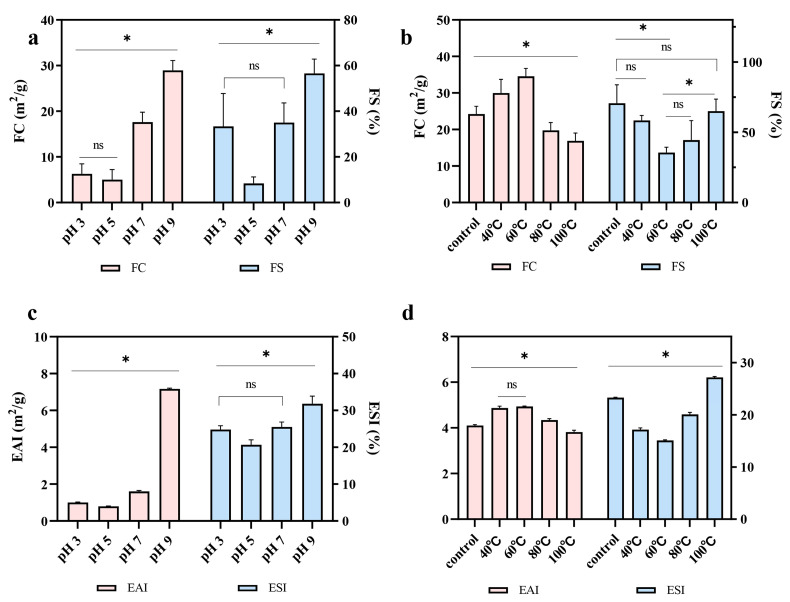
Changes in foaming properties and emulsifying properties of pea albumin at different pH values (3, 5, 7, and 9) and temperatures (40, 60, 80, and 100 °C), (*n* ≥ 3). (**a**) foaming property variation with pH, (**b**) foaming property variation with temperature, (**c**) emulsifying property variation with pH, (**d**) emulsifying property variation with temperature. * indicate a significant difference between samples, *p* < 0.05; ns indicates no significant difference.

The foaming capacity (FC) reflects the ability of proteins to form foam at the air–water interface during agitation, whereas foam stability (FS) indicates the ability of the formed foam to resist gravitational collapse. As shown in Figure 6a, both FC and FS exhibited a U-shaped pattern across the tested pH range, which is consistent with the solubility trend. At pH 5, the lowest FC (5.03 m^2^/g) and FS (33.3%) values were observed, likely due to the reduced solubility and aggregation of proteins near the isoelectric point. In contrast, both FC (28.9 m^2^/g) and FS (56.5%) were highest at pH 9. This enhancement may be attributed to the increased net charge of proteins under alkaline conditions, which promotes protein–water interactions and facilitates the incorporation of albumin molecules into air bubbles [17]. The combination of suitable surface hydrophobicity, smaller particle size, and higher solubility results in the highest FC of albumin at pH 9 [42]. This observation was further supported by Pearson correlation analysis (Figure 7a), which showed a significant negative correlation between FC and particle size (R^2^ = −0.76, *p* < 0.05) and a significant positive correlation between FC and solubility (R^2^ = 0.90, *p* < 0.05). The enhanced FS at this pH may be attributed to strong electrostatic repulsion. Pearson correlation analysis also confirmed a significant negative correlation between FS and Zeta potential (R^2^ = −0.65, *p* < 0.05) (Figure 7a). Consistent with these results, Chavan et al. reported that moisture retention within the protein film surrounding air bubbles, together with sufficient electrostatic repulsion within the foam, plays a crucial role in maintaining FS [43].

As shown in Figure 6b, compared to the control, the FC of albumin increased at moderate temperatures and declined at higher temperatures. Moderate heating promotes partial unfolding of the protein, which exposes hydrophobic residues and improves foaming performance [44]. This result is consistent with the Pearson correlation analysis in Figure 7b, which shows a significant positive correlation between FC and surface hydrophobicity (R^2^ = 1.00, *p* < 0.05). Compared to control and moderate heating, excessive heating induces the formation of large aggregates. These aggregates adsorb more slowly at the air–water interface and resist further unfolding, which reduces molecular flexibility and ultimately impairs FC [45]. Pearson correlation analysis further revealed a significant negative correlation between FC and particle size (R^2^ = −1.00, *p* < 0.05). However, the FS decreased under moderate heating, suggesting that partial unfolding may weaken the ability of albumin to maintain a stable interfacial film.

### 2.9. Emulsifying Properties

The emulsification activity index (EAI) and emulsion stability index (ESI) are key parameters used to evaluate the ability of proteins to adsorb at the oil–water interface and to stabilize emulsions [46]. As shown in Figure 6c, both EAI and ESI exhibited a U-shaped trend across the tested pH range, consistent with the solubility pattern of albumin. When the pH increased from 5 to 9, both indices increased, likely due to the higher solubility and smaller particle size of albumin under these conditions [47]. Pearson correlation analysis further confirmed this observation (Figure 7a), showing a significant positive correlation between EAI and solubility (R^2^ = 0.94, *p* < 0.05) and a significant negative correlation between EAI and particle size (R^2^ = −0.85, *p* < 0.05). In addition, the EAI of albumin treated under alkaline conditions was higher than that of acid-treated samples. This improvement may be attributed to the greater negative charge of albumin at alkaline pH, which modifies electrostatic interactions and promotes a more uniform dispersion of protein molecules within the oil–water system [48]. At pH 9, the increased solubility, reduced particle size, and greater electrostatic repulsion enable albumin to adsorb efficiently to the oil–water interface and stabilize droplets. These functional characteristics make pea albumin suitable for use in plant-based beverages, dairy substitutes, salad dressings, and other emulsion-based food systems that require high stability.

The effect of temperature on the emulsifying properties of albumin is shown in Figure 6d. Moderate heating enhances the EAI, likely because the partially unfolded albumin structure maintains good solubility and surface hydrophobicity. In general, a high EAI requires an appropriate balance of solubility, hydrophobicity, and molecular flexibility [49]. Excessive heating resulted in a marked reduction in EAI. This reduction is likely associated with decreased solubility caused by extensive protein aggregation. Aggregated proteins are less able to unfold rapidly at the oil–water interface, which weakens their interfacial adsorption and emulsifying ability [44]. Pearson correlation analysis also confirms this (Figure 7b), showing a significant positive correlation between EAI and solubility (R^2^ = 0.95, *p* < 0.05). The ESI first decreased and then increased with rising temperature, a trend consistent with the behavior observed for FS.

## 3. Materials and Methods

### 3.1. Materials

Tai Wan Chang Shou Ren pea was provided by Ningxia Pingluo Haofeng Vegetable Seedling Co., Ltd. (Ningxia, China). The BCA protein assay kit was purchased from Beyotime Biotechnology Co., Ltd. (Beijing, China), and soybean oil was obtained from Yihai Kerry Arawana Holdings Co., Ltd. (Beijing, China). NaOH, 1-anilino-8-naphthalenesulfonate (ANS), and other reagents and chemicals were purchased from Solarbio Life Science Co., Ltd. (Beijing, China), and were of analytical grade or higher. Water used was deionized.

### 3.2. Extraction of Pea Albumin

Pea protein was extracted according to the method described by Zhu et al. [50]. Specifically, artificially peeled peas were dried, milled into powder, and dispersed in ultrapure water at a ratio of 1:7 (*w*/*v*). The suspension was magnetically stirred, and 1 M NaOH was added to adjust the pH to 9–10. The mixture was stirred at 25 °C for 2 h and centrifuged at 4000 r/min for 20 min. The supernatant was collected and adjusted to pH 4.5 using 1 M HCl, followed by incubation at 25 °C for 2 h. After centrifugation, the precipitate was collected, washed with distilled water 2–3 times, neutralized to pH 7, and freeze-dried to obtain pea protein powder.

Albumin was subsequently isolated following the procedure of Benimana, with slight modifications [51]. The extraction of albumin was based on differences in protein solubility, as albumin is a water-soluble protein and can be selectively extracted into the aqueous phase. Briefly, lyophilized pea protein was dissolved in ultrapure water at a ratio of 1:10 (*w*/*v*) and subjected to continuous magnetic stirring for 2 h at 25 °C. The resulting suspension was centrifuged at 10,000 rpm for 20 min at 25 °C, and the supernatant was collected. The supernatant was then ultrafiltered through a 30 kDa molecular weight cut-off membrane, and the filtrate was lyophilized to obtain albumin for subsequent analysis.

The albumin fraction was freeze-dried and weighed using an analytical balance with a precision of 0.1 mg. The extraction yield, protein yield, and purity of albumin were subsequently determined, with values of 74.15 ± 1.08%, 7.27 ± 0.11%, and 71.50 ± 0.71%, respectively. The albumin content in raw peas was determined by SDS-PAGE densitometric analysis based on the proportion of albumin bands. Protein purity was measured using the Kjeldahl method, and nitrogen content was multiplied by a factor of 6.25. The calculation methods are described as follows:

Extraction yield (%) = mass of dried albumin obtained/mass of albumin in raw material.

Protein Yield (%) = mass of dried albumin obtained/mass of initial raw material.

### 3.3. pH and Heat Treatments of Albumin

**Figure 8:**
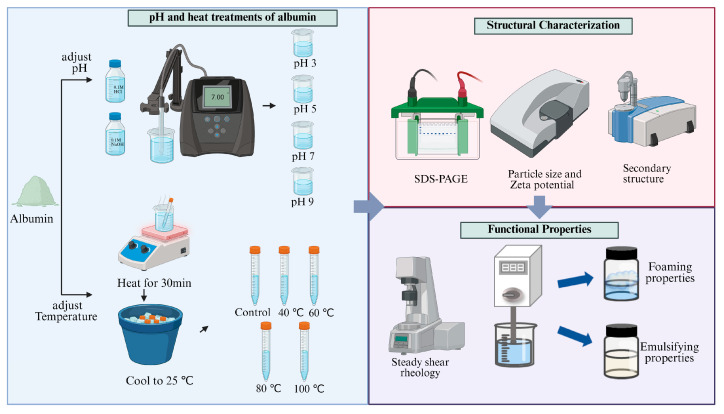
Schematic diagram of the study. Pea albumin was treated under different pH conditions (3, 5, 7, 9) and temperatures (40, 60, 80, 100 °C), followed by structural characterization and functional property measurements.

Albumin was prepared as a 1% (*w*/*v*) protein solution and stirred at 25 °C for 1 h to ensure complete hydration. The solution was then divided into two groups for pH and thermal treatments. For the pH treatment, For the pH treatment, the samples were adjusted to pH 3, 5, 7 and 9 using 0.1 M HCl or NaOH. The pH was measured using a calibrated pH meter and precisely adjusted to the target value within ±0.02 pH units (measured at 25 ± 1 °C). After adjustment, samples were allowed to equilibrate for 30 min prior to subsequent treatments, and the pH was re-checked to confirm stability. The sample at pH 7 was used as the control. For the thermal treatment, the untreated samples with constant pH were placed in a thermostatic water bath and heated at 40, 60, 80, and 100 °C for 30 min. They were then immediately cooled in an ice-water bath to room temperature (25 °C). The processing steps are shown in Figure 8. The unheated samples kept at room temperature (25 °C) served as the control. Some of the treated samples were freeze-dried for further analysis.

### 3.4. SDS-PAGE

The molecular weights of albumin subjected to different treatments were analyzed by SDS-PAGE under reducing conditions. Ten microliters of each protein sample were loaded, and electrophoresis was carried out at a constant voltage. After separation, the gel was stained with Coomassie Brilliant Blue R-250. Protein markers ranging from 11 to 245 kDa and from 3.3 to 20.1 kDa were used as molecular weight standards.

### 3.5. Particle Charge and Size Measurements

The particle size and Zeta potential of protein samples were determined using a Malvern Zetasizer Nano ZS instrument (Malvern Panalytical Co., Ltd., Worcestershire, UK) at 25 °C. Before analysis, samples were diluted in ultrapure water to an appropriate concentration to minimize multiple scattering. Each measurement was conducted using 1 mL of the diluted sample.

### 3.6. Surface Hydrophobicity (H_0_) Measurement

The surface hydrophobicity (H_0_) of pea albumin was determined according to a previously described method [52]. Samples treated under different pH and temperature conditions were diluted to a protein concentration of 0.1–0.5 mg/mL. Subsequently, 25 μL of ANS solution (8 mM) was added to 1 mL of each sample and mixed thoroughly. The fluorescence spectrum was recorded at an excitation wavelength of 390 nm and an emission wavelength of 470 nm. The H0 value of the protein was calculated from the slope of the fluorescence intensity curve.

### 3.7. Secondary Structure Measurement

The secondary structure of pea albumin under different pH and temperature conditions was analyzed using Fourier-transform infrared (FTIR) spectroscopy (Vertex 70, Bruker, Mannheim, Germany) according to the method of Güneş et al. [24]. Protein samples were scanned over a spectral range of 400–4000 cm^−1^, with 16 scans collected at a resolution of 4 cm^−1^. The secondary structures were estimated by second-derivative and Gaussian curve fitting of the amide I band (1600–1700 cm^−1^) using Peak Fit 4.12 software. Secondary structural components were assigned based on their characteristic wavenumber ranges: β-sheet (1610–1640 and 1670–1694 cm^−1^), random coil (1640–1648 cm^−1^), α-helix (1648–1660 cm^−1^), and β-turn (1665–1700 cm^−1^). The relative content of each secondary structural component was calculated from the integrated area of the corresponding sub-peaks, following established protocols [20,53].

### 3.8. Intrinsic Tryptophan Fluorescence Spectroscopy Measurement

The fluorescence spectra of albumin under different pH and temperature conditions were measured using a fluorescence spectrophotometer (FS5, Edinburgh Instruments, Edinburgh, UK), following established protocols [54]. The excitation wavelength was set at 275 nm, with both excitation and emission slit widths fixed at 5 nm. Spectra were recorded over the range of 300–500 nm. Sample solutions were diluted to minimize inner filter effects, ensuring that the measured fluorescence accurately reflected the protein’s intrinsic emission.

### 3.9. Solubility Measurement

The protein content of the samples was determined using the bicinchoninic acid (BCA) assay, following the procedure described previously [34]. A standard curve was prepared using bovine serum albumin (BSA) solutions of known concentrations. The BCA working reagent was prepared by mixing reagents A and B from the commercial kit according to the manufacturer’s instructions. The appropriately diluted samples were transferred into a 96-well microplate and mixed with the working reagent. After incubation at 60 °C for 30 min, the absorbance was measured at 562 nm using an INFINITE E PLEX(CN) microplate reader (Deken Laboratory Equipment Co., Ltd., Shanghai, China). Protein solubility was calculated according to the following equation:

Solubility (%) = Protein content in the supernatant/Total protein content × 100%

### 3.10. Steady Shear Rheology Measurement

The apparent viscosity of pea albumin under different pH and temperature conditions was determined according to a previously described method with minor modifications [35]. According to preliminary experiments, the protein concentration was set at 20% (*w*/*v*). In the linear viscoelastic region (0.5%), measurements were performed using a rotational rheometer (HR-20, TA Instruments, Water, New Castle, DE, USA) equipped with a parallel plate geometry (40 mm diameter). The gap between the plates was set at 5 μm, with a test temperature of 25 °C. Each sample was carefully placed onto the lower plate using a syringe, and any excess material around the geometry was gently removed with a spatula. The samples were equilibrated at 25 °C for 300 s and then subjected to a steady-state scan.

### 3.11. Foaming Properties Measurement

The foaming properties of the protein samples were evaluated according to a previously described procedure with minor modifications [55]. Briefly, protein solutions that had been pretreated under the designated pH and temperature conditions were subsequently homogenized using a high-speed homogenizer (FSH-2, Wuhan Gemolo, Wuhan China) at 10,000 rpm for 2 min to induce foam formation. All volumes were expressed in milliliters (mL), and the foaming capacity (FC) and foam stability (FS) were normalized to the protein concentration of each sample to allow comparison across treatments. The FC and FS were then determined according to the following equations:

FC (%) = (V_t1_ − V_t0_)/V_0_ × 100%

FS (%) = V_t2_/V_t1_ × 100%

where V_t0_ refers to the initial volume of the protein solution before homogenization, V_t1_ represents the total volume of the foamed mixture immediately after homogenization, and V_t2_ indicates the foam volume remaining after a 10 min resting period.

### 3.12. Emulsifying Properties Measurement

The emulsification properties of proteins were determined according to the method of Yan et al. [34]. For each treatment, 15 mL of protein solution at different pH and temperature conditions were mixed with 5 mL of soybean oil. The mixture was homogenized using a high-speed shear homogenizer (FSH-2, Wuhan Gemolo, Wuhan, China) at 10,000 rpm for 2 min. Then, 50 μL of the sample was collected from the bottom of the container and mixed with 5 mL of 0.1% (*w*/*v*) SDS solution, and the absorbance was measured at 500 nm. After standing for 10 min at room temperature (25 °C), another 50 μL sample was taken from the bottom of the container, mixed with 5 mL of 0.1% SDS, and the absorbance was measured again at 500 nm. A 0.1% SDS solution without protein was used as a control. The emulsification activity index (EAI) and emulsion stability index (ESI) were calculated per unit protein concentration (C, g/mL), ensuring that the results reflect the emulsifying performance of protein independently of solution concentration. The emulsification activity index (EAI) and emulsion stability index (ESI) were calculated using the following formulas:

EAI (m^2^/g) = (4.606 × A_t0_ × DF)/(C × φ × 10^4^) (1)

ESI (%) = (A_t0_ × 10)/(A_t0_ − A_t10_) × 100% (2)

In the equations, A_t0_ and A_t10_ represent the absorbance values measured at 0 and 10 min, respectively; C denotes the initial protein concentration (g/mL); and φ represents the oil phase fraction, which was 0.25 in this study; 4.606 represents the interfacial area of the emulsion, which is twice its turbidity (expressed as 2.303 Abs), according to the Mie theory of light scattering.

### 3.13. Statistical Analysis

Sample preparation, treatments, and analyses were performed independently in at least triplicate, results were expressed as the mean ± standard deviation (SD). Statistical analysis was performed using one-way analysis of variance (ANOVA), followed by Duncan’s multiple range test with SPSS software (version 27.0; SPSS Inc., Chicago, USA). Differences were considered statistically significant at *p* < 0.05.

## 4. Conclusions

This study systematically investigated the independent effects of pH and thermal treatments on the structural and functional properties of pea albumin, providing new insights into how environmental factors modulate protein conformation and functionality. The results demonstrated that both factors independently modulate protein conformational behavior. Compared with conditions near the isoelectric point (pH 5), alkaline conditions (pH 9) produced markedly stronger electrostatic repulsion, leading to a 29.5% reduction in particle size, a 76.47% increase in Zeta potential, enhanced protein unfolding, and a 19.06% increase in surface hydrophobicity. Under this pH, solubility improved by 24.8%, accompanied by significant enhancements in foaming and emulsifying properties. Moderate heating at 60 °C induced partial unfolding of pea albumin, reducing particle size by 24.6%, increasing solubility, and improving overall functional performance. However, excessive heating at 100 °C intensified protein–protein interactions, resulting in aggregation, a 14.4% decrease in surface hydrophobicity, and impaired solubility and emulsifying capacity. Pearson correlation analysis revealed significant relationships between structural changes and functional properties, elucidating the structure–function relationship of pea albumin. This study will focus on the use of food-grade denaturing or reducing agents to further modulate pea albumin structure and to evaluate their effects on solubility and in vitro digestibility, synergistic effect of pH and temperature. These findings are novel in systematically characterizing the individual impact of pH and temperature on pea albumin and have practical implications for plant-based food formulation, offering guidance for optimizing beverages, emulsions, and aerated products with improved solubility, foaming, and emulsifying performance. This work provides a theoretical foundation for the rational design and industrial application of pea albumin-based ingredients.

## Data Availability

The original contributions presented in this study are included in the article. Further inquiries can be directed to the corresponding author(s).
